# The Veterinary Medical Association of New Jersey

**Published:** 1900-06

**Authors:** S. Lockwood

**Affiliations:** Secretary


### SOCIETY PROCEEDINGS.

### THE VETERINARY MEDICAL ASSOCIATION OF NEW JERSEY.

The forty-sixth regular meeting of this Association was held at Newark, N. J., on Thursday, May 10, 1900. The President not having arrived, Secretary Lockwood called the meeting to order at 11 A.M., and Dr. William Herbert Lowe, of Paterson, N. J., was nominated and chosen chairman *pro tem.* by acclamation. Roll-call showed that there were twenty-five members present. The minutes of the last meeting were read and approved. President Hurley arrived later and presided.

Dr. Lowe reported that the compilation of the *Veterinary Medical Register of the State of New Jersey* was well under way and would soon be ready for publication. Letters were read from Drs. Leonard Pearson, R. C. Vail, and W. Horace Hoskins.

The veterinary army bill as passed by the United States Senate was unanimously indorsed and resolutions adopted. A committee on army bill, consisting of Dr. Budd, of Orange; Dr. Runge, of Newark, and Dr. Harker, of Trenton, was appointed to go to Washington to secure the support of the Hon. R. Wayne Parker, Chairman of the Military Committee, and our other Representatives in Congress, for the bill. The House of Representatives was asked to concur in the bill as passed by the Senate, without change or amendment.

On motion of Dr. Harker, the Chair appointed a special committee on revision of the Constitution and By-laws, as follows: Dr. William Herbert Lowe, of Paterson, Chairman; Dr. James T. Glennon, of Newark; Dr. Richard Hasbrouck, of Passaic; Dr. T. Earle Budd, of Orange, and Dr. L. R. Hurley, of Hopewell.

All new members present who were elected at the January meeting were each given his certificate of membership upon signing the Constitution and By-laws.

Upon the completion of routine business two interesting papers were read and discussed. The first one was on "Median Neurectomy," by Dr. J. Payne Lowe, of Passaic, who gave a concise and comprehensive account of his experience with the operation, the indications for the operation, and the results obtained. Dr. Lowe's paper will be found elsewhere in this issue.

Dr. Lowe was followed by Dr. S. S. Treadwell, of Englewood, who read a paper on "Navicular Arthritis," and operations for the relief of the same.

After the reading of the papers Dr. James McDonough, of Montclair, delivered an extemporaneous lecture on "Contraction of the Horse's Foot," which was both original and instructive. His remarks showed that he has made his subject a life study, and that his study has been coupled with a large experience and a sound judgment. Several members who heard his remarks said that they had never heard the subject treated in such a practical manner before; that it had even surpassed anything that they had heard in college.

Dr. J. Payne Lowe, upon invitation of the Association, will perform the median neurectomy operation for the relief of chronic tendonitis at the next meeting of the Association, which will be held at Atlantic City on the second Thursday in September. Other essayists for this meeting will be Drs. George W. Pope, E. Matthews, S. R. Sauter, and E. L. Loblein. It is expected that the attendance at this meeting will be very large.

S. LOCKWOOD,
Secretary.

